# Genetic alterations in MED12 promote castration-resistant prostate cancer through modulation of GLI3 signaling

**DOI:** 10.22099/mbrc.2023.47346.1828

**Published:** 2023

**Authors:** Thu Minh Duong, Mariana Araujo Rincon, Nishanth Myneni, Marieke Burleson

**Affiliations:** 1 Department of Biology, University of the Incarnate Word, San Antonio, TX, USA; 2 Department of Molecular Medicine, UT Health San Antonio, San Antonio, TX, USA

**Keywords:** MED12, castration-resistant prostate cancer, SHH signaling, GLI3, tumorigenesis

## Abstract

Prostate cancer is a disease that depends on androgenic stimulation and is thus commonly treated with androgen deprivation therapy (ADT). ADT is highly successful initially; however, patients inevitably relapse at which point the cancer grows independently of androgens and is termed castration-resistant prostate cancer (CRPC). CRPC develops through various mechanisms, one of these being crosstalk of the androgen receptor (AR) signaling pathway with other signaling pathways. Congruently, prior work has shown that androgen deprivation induces SHH signaling, which subsequently promotes activation of AR-dependent gene expression to promote cell growth. Mechanistically, this crosstalk involves a physical interaction between AR and components of SHH signaling, specifically proteins of the GLI transcription factor family. These findings thus suggest that activation of SHH signaling could promote the recurrence of cell growth in the absence of androgens to ultimately lead to progression towards CRPC. In this study, we have investigated this mechanism in a subset of prostate cancer that harbors genetic alterations within the Mediator subunit 12 (MED12). We found that loss of MED12 promotes the expression of GLI3 target genes which subsequently drives excessive cell growth in the absence of androgens. Thus, we conclude that genetic alterations within MED12 promote CRPC through hyperactivated GLI3 dependent sonic hedgehog signaling.

## INTRODUCTION

Prostate cancer remains to be a major health concern among men with estimates that approximately, 288,300 US men will be diagnosed with prostate cancer in 2023 alone and 34,700 of these cases resulting in death [1]. Thus, improved treatment regimens are critically needed for patients suffering from this disease. One standard form of treatment for prostate cancer remains to be androgen deprivation therapy (ADT) as prostate cancer initiation and progression is highly dependent upon circulating androgens [2]. This form of treatment is initially highly successful, however, the majority of patients will eventually succumb to reoccurrence of the disease in the form of castration resistant prostate cancer (CRPC) [3-6]. CRPC is characterized as an advanced form of prostate cancer that does not require the presence of androgens for cell growth. The ability of prostate cancer cells to bypass the androgen requirement is multifactorial, but often includes alterations to the androgen receptor (AR), intratumoral androgen stimulation, or crosstalk with alternate pathways that ultimately promote reactivation of AR [7, 8]. In this current study, we have focused our efforts on the latter mechanism by investigating the involvement of the Sonic Hedgehog (SHH) signaling pathway in progression towards CRPC. 

SHH signaling is a developmental signaling pathway that contributes to the development and adult homeostasis of several major organs, including the prostate [9-11]. SHH signaling is dependent upon nuclear effector proteins of the GLI transcription factor family. Within this family, GLI3 function as a multifunctional regulator with activator and inhibitor functions [12-14]. Importantly, since GLI3 regulates a plethora of genes that play critical roles in cell proliferation pathways, the aberrant activation of SHH signaling has been linked to a number of different cancers [15, 16]. Recent studies have provided insight into the importance of SHH signaling reactivation in CRPC specifically. First, it has been demonstrated that androgen deprivation induces SHH signaling [17-22]. Second, activation of SHH signaling, and subsequent stabilization of full length GLI3, has been shown to promote reactivation of AR dependent gene expression through a mechanism that involves a physical interaction between AR and GLI proteins [18, 19, 21, 23]. Third, enhanced GLI3 protein levels promotes recurrence of androgen independence and subsequent growth of CRPC cells [24]. Thus, it is highly feasible that aberrant activation of the SHH signaling pathway is a key player in causing prostate cancer cells to bypass androgen dependence to promote progression towards CRPC. In this study, we have highlighted the role of the Mediator subunit 12 (MED12) in this process as MED12 is frequently mutated in prostate cancer and has previously been linked to regulation of SHH signaling [25, 26]. 

Mediator is a multi-subunit complex that serves as a bridge between gene-specific transcription factors and RNA polymerase II [27]. One of the subunits within Mediator, MED12, has been shown to be a critical regulator of target genes from several physiological signaling pathways, including SHH signaling [26, 28]. Previous reports have shown that GLI3 and MED12 form a physical connection and that disruption of this interaction leads to hyperactivation of GLI3-dependent SHH signaling [25, 26]. Congruently, pathogenic mutations within MED12 have been linked to X-linked intellectual disability (XLID) syndromes as a direct result of dysregulated GLI3 activity [26]. Interestingly, prostate cancer-associated MED12 mutations lie within close proximity to XLID-linked mutations thus indicating that MED12 mutant prostate cancer likely involves a similar mechanism [29, 30]. Therefore, in this study, we have investigated the link between MED12 and GLI3-dependent SHH signaling and how MED12 mutations promote progression of primary prostate cancer towards CRPC.

## MATERIALS AND METHODS

**Cell culture:** All cells were cultured at 37°C and 5% CO_2 _in RPMI 1640 media supplemented with 10% fetal bovine serum (HyClone) and penicillin-streptomycin-L-glutamine (Invitrogen). Androgen deprived media was comprised of RPMI 1640 media without phenol red supplemented with 10% charcoal stripped fetal bovine serum and penicillin-streptomycin-L-glutamine (Invitrogen). 

**Generation of MED12 knockdown cell line: **pLKO.1 vector containing no shRNA (shCT) or MED12 shRNA together with psPAX2 and pMD2.G (Invitrogen) were transfected into HEK293T cells. Viral particles were harvested by ultracentrifugation at 26K and used for subsequent infection of LNCaP cells. Infected cells were selected with 3 µg/ml puromycin. 

**Western blot analysis: **For all western blot analyses, whole cell lysates were prepared and 100 µg total protein was resolved on 10% SDS-PAGE. Protein was detected using the following antibodies at indicated dilution: anti-MED12 (Invitrogen PA5-51852@1:1000 dilution), anti-GLI3 (Invitrogen PA5-28029@1:1000 dilution), and anti-TFIIEµ (Invitrogen 11596-1-AP@ 1:3000 dilution).

**Quantitative real time PCR:** Cells were seeded at 4×10^5^ cells in 60mm dishes in 4 ml of either androgen replete or androgen deprived media and regularly split when ~80% confluent. RNA was extracted on day 10 using Trizol reagent. Oligo(Dt) and superscript III (Invitrogen) were used to reverse transcribe the RNA following standard procedures and was subsequently used for quantitative PCR. 

**Proliferation assays: **Cells were seeded at 1×10^4^ cells per well in 6 well plates in triplet repeats using 2 ml of either androgen replete or androgen deprived media. Cells were harvested on indicated days using standard trypsin protocols and counted on a hemocytometer. For double knockdown, stable MED12 knockdown cells were infected with GLI3-specific shRNA and selected as previously described prior to cell seeding.

**Colony formation assays: **Cells were seeded at 4000 cells/well in a 6-well plate using 2 ml of either androgen replete or androgen deprived media. Media was changed every 3 days and knockdown cells were kept at 3 µg/ml puromycin selection. On day 15, cells were washed with PBS, fixed with 10% paraformaldehyde and stained with 0.1% crystal violet. For double knockdown, stable MED12 knockdown cells were infected with GLI3-specific shRNA and selected as previously described prior to cell seeding. 

## RESULTS

**Figure 1:**
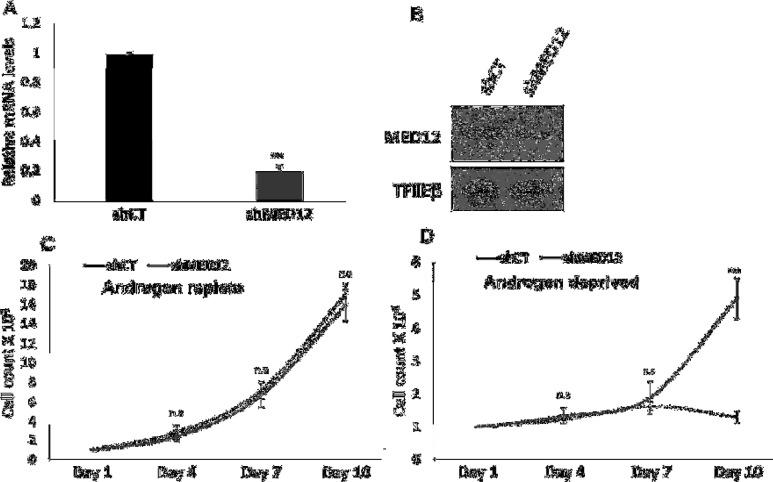
MED12 knockdown promotes enhanced cell proliferation in the absence of androgens.

Prior studies have shown that XLID-linked MED12 mutations lead to inactivation of MED12 protein function which subsequently promotes dysregulation of GLI3 signaling [26] Since the mutations found in XLID syndromes closely cluster to the prostate cancer associated MED12 mutations we presume that these mutations similarly lead to inactive MED12 protein function. Thus, we simulated the prostate cancer MED12 mutant setting by generating a lentivirus mediated stable MED12 knockdown LNCaP cell line (Fig 1 A, B). Proliferation assays were performed on the MED12 knockdown cells to determine whether depletion of MED12 promotes hyperproliferation of prostate cancer cells. Interestingly, we show that reduced MED12 expression does not significantly affect prostate cancer cell growth in androgen replete conditions, whereas we observed a significant increase in cell proliferation in androgen deprived conditions (Fig 1 C, D). Therefore, we conclude that MED12 expression is a key player in regulating prostate cancer cell growth specifically in the absence of androgens. These results thus provide a possible link between MED12 genetic alterations and progression to CRPC in patients post-ADT. 

**Figure 2:**
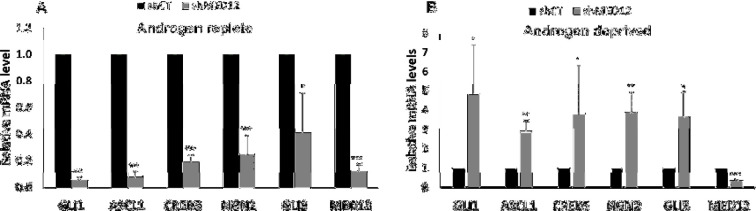
GLI3 target genes are upregulated in MED12 knockdown cells in absence of androgens.

To determine the mechanism underlying increased cell proliferation in the MED12 knockdown setting, we investigated the involvement of SHH signaling. Since prior studies have shown that MED12 modulates GLI3-dependent SHH signaling [25, 26], we focused our efforts towards examining the effect of MED12 knockdown on the expression of GLI3 target genes. Interestingly, we show that GLI3 target genes are downregulated upon MED12 knockdown in the presence of androgens, whereas we observe a significant upregulation in the absence of androgens (Fig. 2 A, B). Nonetheless, these results are consistent with MED12 placing a constraint on GLI3-dependent SHH signaling in cells that have been deprived of androgens. When MED12 is either mutated or downregulated this constraint gets released thereby promoting increased expression of GLI3 target genes which could subsequently cause progression towards CRPC. 

A) Confirmation of MED12 knockdown in LNCaP cells by qRT-PCR using primers against MED12. mRNA levels are expressed relative to the level in shControl (shCT) cells. Data is mean +/- SEM from 3 replicate assays. B) Confirmation of MED12 knockdown in LNCaP cells via western blot analysis. C and D) Proliferation assays of MED12 knockdown cells in androgen replete and androgen deprived conditions. Data is mean +/- SEM from 3 replicate assays. Asterisks denote statistically significant difference (Unpaired two-tailed student t test: *** P<0.001).

A and B) qRT-PCR of shControl (shCT) or shMED12 LNCaP cells in androgen replete or androgen deprived conditions respectively. mRNA levels were normalized to β-actin mRNA and expressed relative to their levels in shCT cells. Data is mean +/- SEM from 3 replicate assays. Asterisks denote statistically significant difference (Unpaired two-tailed student t test: * P<0.05, ** P<0.01, *** P<0.001).

**Figure 3:**
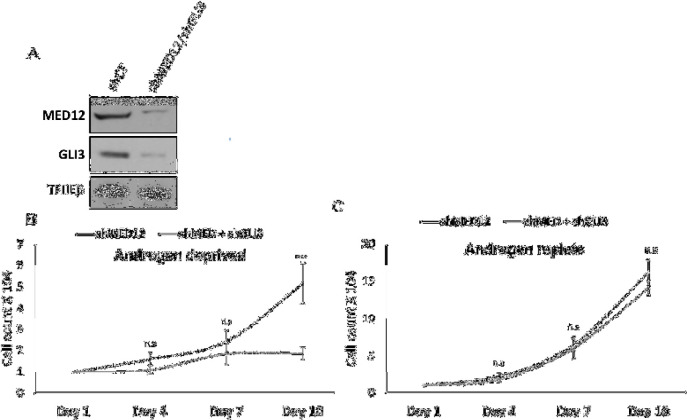
Enhanced cell proliferation of MED12 knockdown cells is dependent on GLI3.

**Figure 4:**
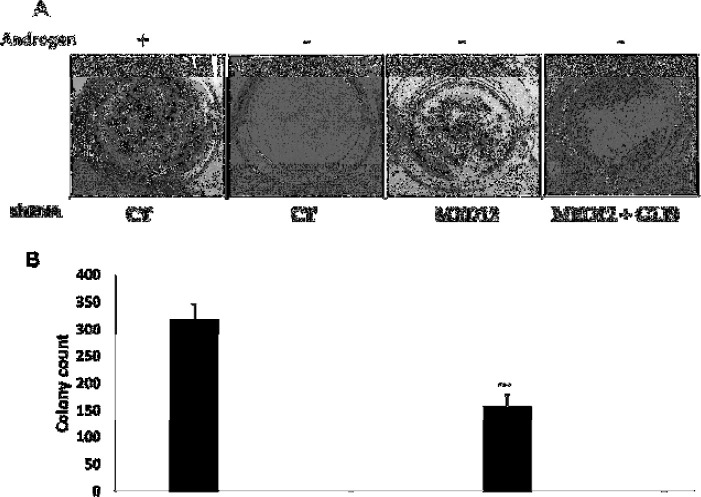
In vitro colony formation of MED12 knockdown cells is dependent on GLI3.

Since we showed that loss of MED12 leads to increased cell proliferation in the absence of androgens, we next wanted to determine whether GLI3 is required for this process. To do so, we performed proliferation assays in cells that were simultaneously knocked down for MED12 and GLI3 (Fig. 3A). We observed that enhanced cell proliferation of MED12 knockdown cells in the absence of androgens is reversed upon double knockdown with GLI3 (Fig. 3B). Importantly, knockdown of GLI3 does not significantly affect the proliferation of MED12 knockdown cells in the presence of androgens (Fig. 3C). To further support these findings, we performed colony formation assays in single and double knockdown cells. Consistent with our previous data, we observed that colony formation is fully blocked upon androgen deprivation and that knockdown of MED12 partially rescues the ability to form colonies (Fig 4 A, B). Importantly, double knockdown of MED12 and GLI3 leads to a complete lack of colony formation in androgen deprived conditions (Fig 4 A, B). These results together thus further support the involvement of GLI3-dependent SHH signaling in promoting cell proliferation and subsequent tumor formation of MED12-altered prostate cancer cells that have undergone ADT.

A) Confirmation of double knockdown via western blot analysis. B and C) Proliferation assay of single (shMED12) or double (shMED12 + shGLI3) knockdown LNCaP cells. Data is mean +/- SEM from 3 replicate assays. Asterisks denote statistically significant difference (Unpaired two-tailed student t test: *** P<0.001).

A) Colony formation assay of shCT, shMED12, or shMED12 + shGLI3 LNCaP cells grown in either the presence or absence of androgen. B) Quantification of 4 replicate colony formation assays +/- SEM. Asterisks denote statistically significant difference (Unpaired two-tailed student t test: *** P<0.001).

## DISCUSSION

**Figure 5:**
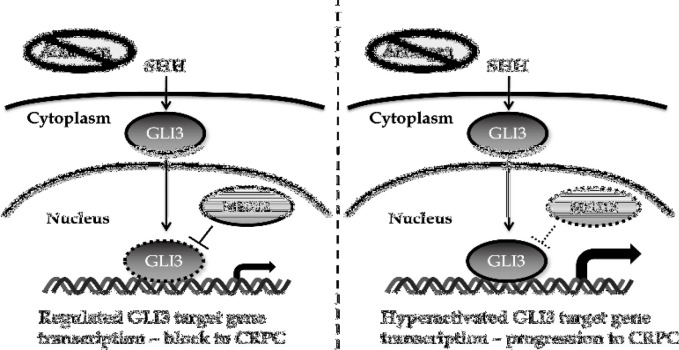
Schematic diagram of working model. In the absence of androgen, the SHH signaling pathway is turned on leading to an upregulation of the downstream effector GLI3. In the presence of wildtype MED12 (left), a constraint is placed on GLI3 signaling thereby leading to regulated GLI3 target gene expression and a subsequent block to progression towards CRPC. When MED12 is either downregulated or mutated (right), the constraint on GLI3 is lifted thereby leading to hyperactivated GLI3 target gene expression and subsequent progression to CRPC.

Our findings have uncovered a novel role of MED12 in the progression of primary prostate cancer towards CRPC. We found that loss of MED12, through lentiviral-mediated knockdown, caused a significant increase in cell proliferation in androgen deprived conditions. Furthermore, there was no effect on cell proliferation when cells were grown in the presence of androgens. Congruently with this finding, MED12 knockdown cells grown in the absence of androgens, but not in the presence of androgens, showed a significant reduction in the ability to form colonies in vitro. Mechanistically, we found that the GLI3-dependent SHH signaling pathway played an important role in promoting cell growth through two key findings. First, we showed that GLI3 target genes are significantly upregulated in MED12 knockdown cells grown in the absence of androgens. Secondly, we showed that the enhanced cell growth and colony formation ability of MED12 knockdown cells is dependent on GLI3 as GLI3 knockdown reverses the enhanced cell growth observed due to MED12 knockdown. Overall, these findings lead us to our working model (Fig. 5). Previous research has shown that androgen deprivation induces SHH signaling which subsequently activates GLI3 [17-22]. Under wildtype conditions, MED12 would place a constraint on GLI3 thereby preventing hyper-activation of GLI3 target genes. When MED12 is either mutated or downregulated, the constraint on GLI3 gets released thereby inducing expression of GLI3 target genes and subsequent cell proliferation and progression towards CRPC (Fig. 5). Ultimately, these findings provide critical insight into the mechanisms underlying CRPC development for MED12-mutant prostate cancer. Based on our findings, the use of SHH inhibitors could prove to be beneficial in treating this subset of prostate cancer. Furthermore, the development of novel GLI3 inhibitors can provide an avenue for a highly targeted approach for patients suffering from this disease.

## Conflict of Interest:

The authors declare no conflicts of interest
